# Survival Rate of Zygomatic Implants for Fixed Oral Maxillary Rehabilitations: A Systematic Review and Meta-Analysis Comparing Outcomes between Zygomatic and Regular Implants

**DOI:** 10.3390/dj9040038

**Published:** 2021-04-01

**Authors:** Felice Lorusso, Roberto Conte, Francesco Inchingolo, Felice Festa, Antonio Scarano

**Affiliations:** 1 Department of Innovative Technologies in Medicine & Dentistry and CAST, University of Chieti-Pescara, Via dei Vestini 31, 66100 Chieti, Italy; drlorussofelice@gmail.com (F.L.); felice.festa@unich.it (F.F.); 2 Private Practice, 35010 Padova, Italy; dott.roberto-conte@libero.it; 3 Department of Interdisciplinary Medicine, University of Bari “Aldo Moro”, 70121 Bari, Italy; francesco.inchingolo@uniba.it

**Keywords:** zygomatic implant, endosseous implants, bone ridge atrophy, maxillary fixed rehabilitations

## Abstract

Background: Zygomatic implants have been proposed alone or in combination with premaxillary conventional implants for severe resorbed maxillary atrophy rehabilitation. The aim of the present investigation was to evaluate through a qualitative systematic review and meta-analysis the survival rate of zygomatic implants in conjunction with regular fixtures for maxillary rehabilitation. Methods: The article screening was conducted on the PubMed/Medline and EMBASE electronic databases according to the “Preferred Reporting Items for Systematic Reviews and Meta-Analyses” (PRISMA) guidelines. The scientific papers were included for qualitative analysis and risk-of-bias evaluation. Only the papers that included rehabilitation with zygomatic implants in combination with regular implants were considered for the meta-analysis comparative evaluation of the implant survival rate. Results: The paper search screened a total of 137 papers. After the initial screening, a total of 32 articles were considered for the qualitative analysis. There was a similar implant survival rate between zygomatic and premaxilla regular implants (*p* = 0.02; Z: 2.26). Conclusions: Zygomatic and conventional implants showed a high long-term survival rate for fixed maxillary rehabilitations, but few included studies reported the marginal bone loss after loading. Further studies are necessary to evaluate the pattern of marginal bone loss between zygomatic and conventional implants after long-term functional loading.

## 1. Introduction

The rehabilitation of severely atrophic maxilla represents a complex treatment due to functional and aesthetic alteration related to tooth loss and extreme bone ridge resorption [1,2,3]. Moreover, the loss of masticatory and phonetic efficiency could produce important implications for social relationships and quality of life [4,5]. The positioning of implants to rehabilitate partial or total edentulous ridges represents a validated long-term treatment option, while the availability of adequate bone volume and density could determine a possible clinical limitation for implant fixation and loading [6,7,8,9,10,11]. The atrophies of the maxilla have been classified from Cawood–Howell Class I to Class VI [2]:

- Class I: teeth present.
- Class II: immediate post-extraction socket.
- Class III: edentulous ridge with adequate height and width of bone.
- Class IV: knife-edge ridge, adequate bone height but inadequate in width.
- Class V: flat bone ridge, bone inadequate in width and height.
- Class VI: depressed-form ridge, basal bone resorption.

According to local anatomy and sinus cavity dimensions, many different approaches have been proposed for maxillary rehabilitations with 4/6 tilted dental implants [12,13,14,15] in combination with single/bilateral zygomatic implants. These severe cases are often correlated in clinical practice after severe bone resorption, local infections and resective oncologic surgery [16,17]. Zygomatic implants have been proposed as a valuable treatment option for fixed rehabilitation in severe reabsorbed bone ridges due to the reduced invasivity, morbidity and decreased time required to finalize the rehabilitation [5,17,18]. Zygomatic implant positioning is an approach that avoids grafting or maxillary sinus augmentation and consequently produces a shorter and more comfortable post-operative morbidity [18,19,20]. In fact, the restoration of severe maxillary atrophies often requires an extensive grafting approach with autologous bone from a calvaria, iliac crest or mandibular graft with an higher surgical morbidity, cost of rehabilitation time and a reduced predictivity [21,22,23].

Other indications for zygomatic implants include the previous failure of conventional implant placement, failure of grafting procedures and tumor resection or trauma [4,12,17,24].

The zygomatic bone is a bilateral, pyramidal bone characterized by a cortical and trabecular component. Tomographic studies reported that no significant morphological and volumetric alterations of this region are associated with tooth loss and jaws atrophies while the zygomatic bone has a sufficient bone density and is a candidate for dental implant positioning [25,26,27].

Anatomically, the morpho-structural maintaining of this region is determined by the action of masseter muscles that induce constant bone remodeling stimulation and functional activity [27,28].

Maxillary rehabilitations with four bilateral zygomatic implants or in combination with two or more regular premaxillary implants have been successfully proposed for two-stage or immediate functional loading protocols [29,30,31]. The purpose of the present investigation was to compare the survival rate of combined zygoma maxillary and zygomatic implants in association with regular implants in the premaxilla through a systematic review and meta-analysis.

## 2. Materials and Methods

### 2.1. Database Search Strategy

The PICO (population, intervention, comparison, outcome) question has been reported in Table 1.

The paper’s search and inclusion and study data presentation were performed in accordance to the “Preferred Reporting Items for Systematic Reviews and Meta-Analyses” (PRISMA) guidelines [32]. The more appropriate medical search terms (MeSH) and keywords were identified by the Cochrane library to create a detailed search strategy (Table 2).

The paper’s initial screening was conducted on the PubMed and EMBASE electronic databases (13 December 2020) according the Boolean search detailed in Table 2. The abstracts of scientific studies selected were limited to only human randomized and non-randomized clinical trials, prospective and retrospective studies with zygomatic maxillary rehabilitation and were selected for a full-text evaluation.

### 2.2. Inclusion and Exclusion Criteria

For the qualitative analysis, the inclusion criteria were human clinical trials, prospective and retrospective studies with a minimum follow-up of 6 months. The inclusion criteria were articles describing zygomatic implants for partial or full fixed maxillary rehabilitations with no restrictions on number of zygomatic and regular implants positioned or immediate/delayed loading protocol. The exclusion criteria were systematic reviews, letters to the editor, case reports and case series, in vitro and laboratory simulations and dental implants associated to a bone regeneration/sinus augmentation procedure.

### 2.3. Papers Selection Procedure

The selection of the research papers eligible for the qualitative analysis was performed independently by two reviewers to evaluate the studies’ titles and abstracts. Moreover, a manual search was performed to increase the pool of the studies eligible for full-text evaluation. The screening phase of the papers’ selection included clinical trials with no restrictions about randomization and blinding assessments in order to increase the item pool. The papers written in English that satisfied the inclusion criteria were included, and the full-text was obtained and evaluated. The duplicates and excluded papers were also recorded and categorized according to reasons of review exclusion.

### 2.4. Study Assessment

The study data of the selected papers were recorded and evaluated independently through a specially designed form according to the following categories: study model design, treated patients, number of zygomatic and regular implants, smokers, immediate or delayed zygomatic loading protocol, type of prosthesis, study follow-up, zygomatic and regular implant survival rate, complications and quantity of zygomatic implant failure.

### 2.5. Risk of Bias Assessment

The risk of bias assessment was performed by the software package RevMan 5.5 (The Nordic Cochrane Centre, The Cochrane Collaboration, Copenhagen, Denmark, 2014). The risk of bias evaluation was according to the following parameters and criteria: randomization sequence, allocation concealment, blinding assessment, completeness of procedure description, clearness of inclusion criteria, attrition bias, reporting bias, follow-up length and other bias. The risk of bias criteria were categorized as adequate, unclear or inadequate. The selected studies were categorized as low risk of bias with a minimum ratio of 6/9 positive parameters and an absence of a negative outcome. Otherwise, the research was categorized as high risk.

### 2.6. Comparative Meta-Analysis

The research data were carefully analyzed through a special designed database in Excel (Microsoft, Redmond, WA, USA). The comparative meta-analysis of survival rate was performed including the clinical papers with zygomatic implants in combination with regular implants for fixed maxillary rehabilitations. No limits regarding follow-up, prosthesis typology, quantity of implants or patient characteristics were considered for the evaluation. The means were considered for dichotomous data considering the number of implants with events and the total number of participants in experimental and control groups, while the survival rate of zygomatic implants compared to regular implant groups was considered the study outcome variable. The statistical comparison evaluated the survival rate of zygomatic implants positioned for anchorage in the zygomatic bone vs. regular dental implants positioned in the maxillary alveolar bone. Pterygoid implants, trans-sinus implants and implants positioned in the vomer/nasal crest have been excluded from the present evaluation.

## 3. Results

### 3.1. Paper Selection Process

**Figure 1:**
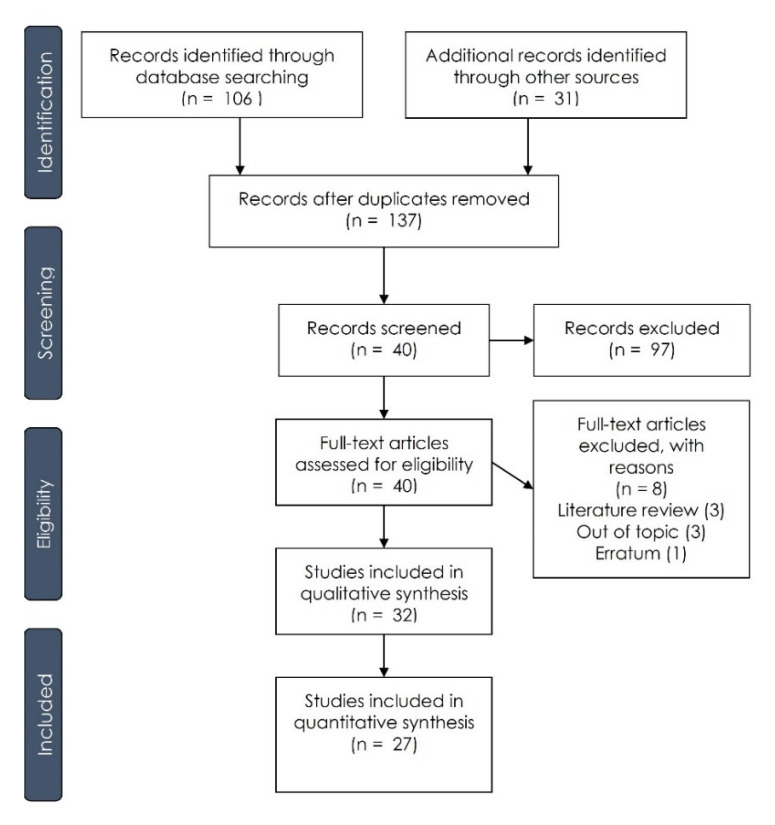
Summary of the manuscript selection process according to the “Preferred Reporting Items for Systematic Reviews and Meta-Analyses” (PRISMA) guidelines.

The manuscript identification, screening, eligibility and inclusion process is presented in Figure 1. The total output list retrieved a total of 137 manuscripts: 106 were identified through the electronic search and 31 were selected by a manual search. After a title and abstract evaluation, a total of 97 manuscripts were excluded after the screening phase, and 40 papers were included for full-text evaluation. A total of 8 full-text papers were excluded: 3 literature reviews, 4 manuscripts that were out of topic and 1 case report. A total of 32 papers were included for the qualitative synthesis [33,34,35,36,37,38,39,40,41,42,43,44,45,46,47,48,49,50,51,52,53,54,55,56,57,58,59,60,61,62,63,64], and 27 articles were considered for the meta-analysis [33,34,35,36,37,38,39,40,41,42,43,44,45,46,48,50,51,53,54,55,56,57,59,60,61,63,64].

### 3.2. General Property of the Studies Included

The articles were described according to the zygomatic implant positioned for each rehabilitation, type of prosthesis, follow up time, implant survival rate, surgical complications and zygomatic implants failed. The main characteristics of the studies included were described in Table 3 and Table 4 according to study model, patients, number of regular and zygomatic implants, loading, prosthetic rehabilitation, study outcome and follow-up time. A total of 17 retrospective studies and 15 prospective studies were retrieved from the search.

### 3.3. Study Characteristics and Risk of Bias Assessment

**Figure 2:**
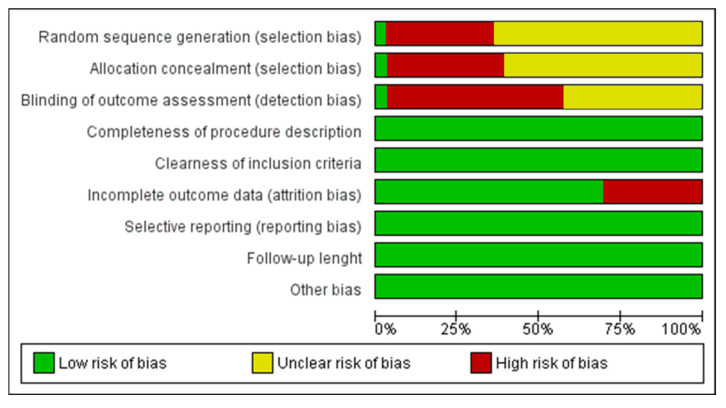
Summary of the risk of bias assessment of the studies included.

**Figure 3:**
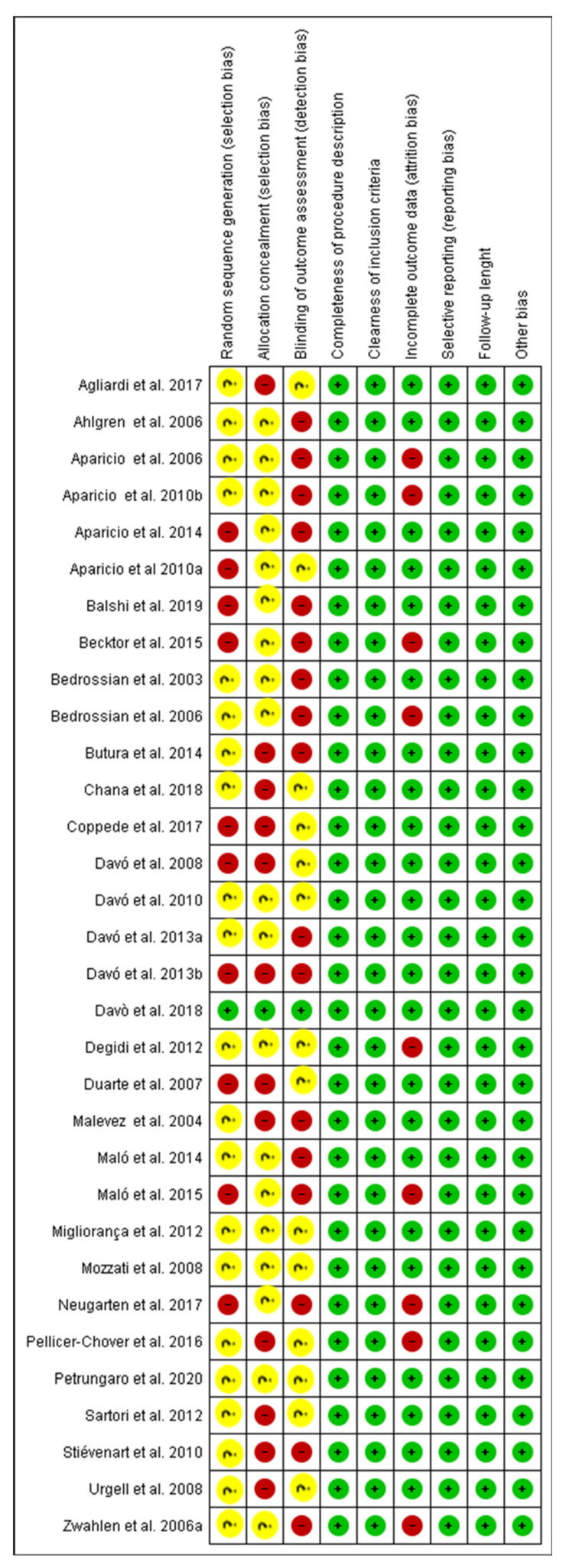
Details of the risk of bias assessment of the studies included.

The patients’ recruited pool ranged from 7 to 352 subjects (mean: 44.68 ± 70.14) and 14–747 zygomatic implants (mean: 96.96 ± 140.85) positioned. The range of regular implants positioned was 18–795 screws (mean: 154.96 ± 163.26). The patients’ recruited pool ranged from 7 to 352 subjects (mean: 44.68 ± 70.14) and 14-747 zygomatic implants (mean: 96.96 ± 140.85) positioned. The range of regular implants positioned was 18–795 screws (mean: 154.96 ± 163.26). A total of 259 single zygomatic implant rehabilitations (mean: 18.50 ± 38.01), 798 double zygomatic implant rehabilitations (mean: 34.70 ± 43.32), 23 triple (mean: 5.75 ± 5.73) and 211 quadruple zygomatic implant rehabilitations (mean: 16.23 ± 15.36). A total of 1348 full-arch prostheses (mean: 43.48 ± 69.85) and 27 partial fixed prostheses (mean: 6.75 ± 5.68) were evaluated for the qualitative analysis. The analysis of the risk of bias of the papers included is presented in Figure 2 for a total of 32 papers. A total of 5 papers were considered to have a low risk of bias (Figure 2 and Figure 3) with a wide heterogeneity of study model design, type of rehabilitation and functional loading follow-up period. Davò et al. al. was the only randomized and blinded clinical study included for the qualitative evaluation and meta-analysis comparison [46].

### 3.4. Meta-Analysis Evaluation

**Figure 4:**
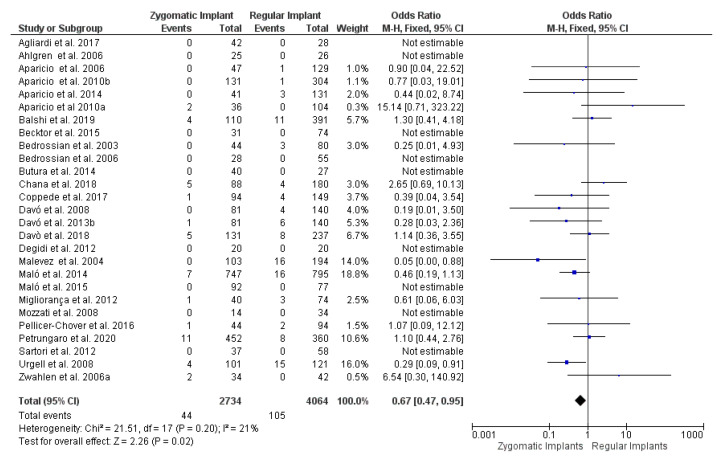
Forest plot of comparison between the zygomatic and regular implant survival rate.

**Figure 5:**
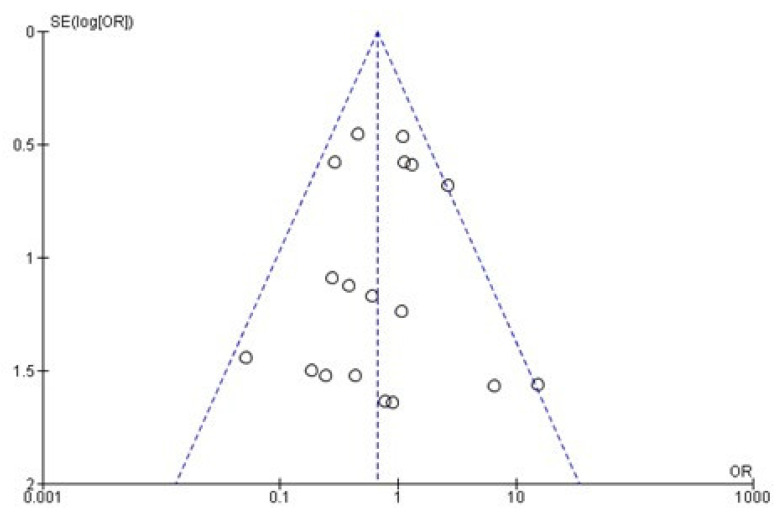
Funnel plot of the zygomatic and regular implant survival rate comparation.

After the qualitative analysis, a total of 27 articles were selected for the comparative evaluation of meta-data of the zygomatic vs. regular implant survival rate. For the meta-data evaluation, papers were considered with a minimum of 6 months follow-up with a zygomatic and regular implant-combined fixed rehabilitation. The minimum follow-up period of the selected paper was 6 months, and the maximum was 97 months. The analysis showed a significant overall effect [*p* = 0.02; Z: 2.26]; heterogeneity [*p* = 0.20; χ2: 21.51, df: 1; I^2^: 21%]. The odds ratio (OR) was 0.67 (95 CI: 0.47–0.95) (Figure 4 and Figure 5).

## 4. Discussion

The present investigation aimed to evaluate through a qualitative analysis the effectiveness and survival rate of regular vs. zygomatic implants for combined fixed maxillary rehabilitation in the literature through a meta-analysis. In the present study, a significantly higher survival rate of zygomatic implants vs. regular maxillary implant was present, while included papers showed a wide heterogeneity of study design, surgical protocols with or without bone graft and regeneration procedures and implant geometries. Reasonably, the survival rate of both regular and zygomatic implants could be influenced in a decisive manner by all of the previously described factors. Moreover, the implant loading protocols [8,65,66], the different sizes [67,68,69,70], prosthetic emergence profiles [71,72], the number of zygomatic implants positioned [29,30], the different loading angle of zygomatic compared to regular implants [28,43,73,74] and the quantity of keratinized tissues [75,76] could represents key factors for the long terms maintenance of soft and hard tissue levels [38,77]. Moreover, the positioning localization of the implant screws could also play an important role. In fact, the masticatory forces are dissipated in a more apical position by the zygomatic implants at the level of the malar prominence of the maxillary bone if compared to conventional screws, where they are discharged at the level of the maxillary bone ridge at a functional distance from the aggression of bacteria biofilm adhesion and infection risk factors present in the oral mouth [28,74].

Gümrükçü et al. investigated the biomechanics of bilateral zygomatic implant configurations for full maxillary rehabilitations, measuring the stresses and deformation of the skull bone with a 150 N vertical occlusal and 300 N masseteric loading [74]. The authors concluded that the maximum von Mises stress was reported in type 4 defects and D3 bone types, while the minimum stress was reported in type 1 buccal bone defects and D2 bone types [74]. In the literature, it was reported that an important role was determined by the zygomatic bone support for the biomechanics and survival of zygomatic rehabilitation [28].

Romeed et al. reported that a residual zygomatic bone support of 10 mm was correlated to a significant increase of zygomatic implant biomechanical stress [28]. The authors evidenced that the zygomatic fixture deflection was lower than 2/3 times in the case of zygomatic residual bone support of 15/20 mm [28]. Moreover, the study evidenced that the von Mises stress (MPa) under occlusal loading was dissipated at the level of the bone-implant interface and no significant difference were reported in the region of the abutment prosthetic joint [28].

Zygomatic and regular implants showed survival rate ranges of 94.1–100% and 91.25–100%, respectively, while the most common early complications (<6 months from the procedure) for zygomatic implant procedures were osteointegration failure, sinusitis, Schneiderian membrane perforation, implant mobility, pain, eye orbit drill penetration and oro-antral fistula (Table 2). Moreover, the literature has reported rare complications such as aspergillosis associated to fungus contamination during the surgery and intracerebral penetration, while a little error in the of angle of the implant site preparation could determine an invasion of critical anatomic regions [30]. The delayed most common complication represented is essentially the sinusitis that could occur years after the surgery [30].

Few articles screened additionally reported the marginal bone loss values around zygomatic and regular implants [33,45]. Agliardi et al. reported on nine full restorations with double bilateral zygomatic implants and six full rehabilitations with quadruple zygomatic implants; after a minimum follow-up of 6 years, there was a mean bone loss for regular implants of 1.39 ± 0.10 mm and a mean bone loss for regular implants of 1.36 ± 0.09 mm with no significant differences between the two groups [33].

Davò et al. reported in a patient with a quad zygomatic for fixed maxillary rehabilitation the failure of a total of three zygomatic implants after 3 weeks [46]. In this particular case, the subject changed the rehabilitation planning into a removable overdenture [46].

Coppedè et al. reported after a 3-year clinical prospective follow-up 1.34 ± 0.23 mm mean bone loss for zygomatic implants and 1.10 ± 0.58 mm mean bone loss for regular implants [45]. Probably, more long-term evaluations and histological studies should be considered for future research to highlight the comparative responses of the peri-implant tissues around zygomatic and conventional implants after functional loading.

### Limitations of the Study

The length, diameter and macro- and microgeometry of the fixtures are determinant for the successful osteointegration of zygomatic and regular implants [28,78,79]. No limits regarding size, surface treatments, implant geometry, surgical approaches, techniques and eventual regenerative procedures were imposed for the article screening. Moreover, the differences regarding prosthesis design, the research and the follow-up model could have a strong influence on the study’s effectiveness. Another important limitation of the study is that no randomized and blinded studies were included in the present investigation, which could represent a determinant bias for the statistical considerations.

## 5. Conclusions

In conclusion, zygomatic implants are a long term predictable option for severe maxillary atrophies treatment with combined zygomatic fixed implant-supported rehabilitations, showing a higher cumulative survival rate compared to conventional implants. More future clinical trials and histological studies on retrieved biopsies are required to evaluate the long term effectiveness of peri-implant soft and hard tissue response around zygomatic and conventional implants after loading.

## Figures and Tables

**Table 1:** The PICO (population, intervention, comparison, outcome) question.

Population\Patients	Intervention	Comparison	Outcomes
Patient group of interest?	What is the main intervention you wish to consider?	Is there an alternative intervention to compare?	What is the clinical outcome?
Subjects that need oral rehabilitation with zygomatic implant surgery in maxillary severe atrophies.	Zygomatic implant positioning for fixed maxillary rehabilitation.	Zygomatic implant vs. regular maxillary implant survival rate.	Can zygomatic implants provide a valuable survival rate for fixed rehabilitation in maxillary severe atrophies?

**Table 2:** Electronic database Boolean search: keyword strategy.

	Search Strategies
Keywords:	Advanced keyword search: (zygomatic dental implant AND oral rehabilitation) AND (retrospective study OR prospective study OR controlled study)
Databases	PubMed/Medline, EMBASE

**Table 3:** Summary table of the papers included for the qualitative evaluation.

Authors	Year	Journal	Zygomatic Implant Configuration	Prosthesis	Follow up	Zygomatic-Regular Implant Survival Rate	Complications	Zygomatic Implant Failure
Agliardi et al.	2017	Int J Oral Maxillofac Surg	Double (9) Quad (6)	Full-arch (15)-Partial (0)	79 m to 97 m	100%-/	-	-
Ahlgren et al.	2006	Int J Oral Maxillofac Implants	Double (13)	Full-arch (13)-Partial (0)	11–49 m	100–100%	-	-
Aparicio et al.	2010	Clin Implant Dent Relat Res	Single (4) Double (17)	Full-arch (20)-Partial (0)	36–48 m	100–100%	-	-
Aparicio et al.	2006	Clin Implant Dent Relat Res	Single (7) Double (62)	Full-arch (69)-Partial (0)	60 m	100–99%	Sinusitis	-
Aparicio et al.	2010	Clin Implant Dent Relat Res	-	Full-arch (23)-Partial (2)	24 m to 60 m	100–99.2%	Regular implant failure (1), Abutment screw fracture (5)	1 implant
Aparicio et al.	2014	Clin Implant Dent Relat Res	Single (3) Double (41)	Full-arch (22)-Partial (0)	120 m	95.12–97.71%		2 implants
Balshi et al.	2009	Int J Oral Maxillofac Implants	-	Full-arch (56)-Partial (0)	9–70 m	96.37–97.2%	Osseointegration failure	4 implants
Becktor et al.	2005	Clin Implant Dent Relat Res	Single (1) Double (15)	Full-arch (16)-Partial (0)	9–69 m	100%	Sinusitis	-
Bedrossian et al.	2006	Int J Oral Maxillofac Implants	Double (14)	Full-arch (14)-Partial (0)	6 m	100–100%	-	-
Bedrossian et al.	2003	Int J Oral Maxillofac Implants	Double (22)	Full-arch (22)-Partial (0)	34 m	100–91.25%	-	-
Butura et al.	2014	Int J Oral Maxillofac Implants	Single (1) Double (12), Triple (1), Quad (1)	Full-arch (15)-Partial (0)	24 m	100–100%	-	-
Chana et al.	2018	Int J Oral Maxillofac Implants	-	-	216 m	94.32%	Sinusitis, implant mobility, pain	5 implants
Coppede et al.	2017	Clin Implant Dent Relat Res	Single (6) Double (27), Triple (3), Quad (6)	Full-arch (42)-Partial (0)	36 m	98.9–97.7%	Osseointegration failure	1 implant
Davò et al.	2018	Eur J Oral Implantol	Double (6) Quad (29)	Full-arch (35)-Partial (0)	12 m	96.1–91.6%	Sinus membrane perforation (4), major swelling (1), sinusitis (4), implant mucositis (2), Peri-orbital infection and swelling (2)	5 implants/3 patients
Davó et al.	2013	Eur J Oral Implantol	-	Full-arch (37)-Partial (5)	60 m	98.5–94.9%	Osseointegration failure, peri-implant mucositis	1 implant
Davó et al.	2010	Eur J Oral Implantol	Quad (17)	Full-arch (17)-Partial (0)	12 m	100–100%	Eye orbit drill penetration, fistula	-
Davó et al.	2008	Eur J Oral Implantol	Single (5) Double (35) Quad (2)	Full-arch (37)-Partial (5)	12-42 m	100–97%	Implant mobility	-
Davó et al.	2013	Eur J Oral Implantol	Quad (17)	Full-arch (17)-Partial (0)	36 months	100%-/	Eye orbit drill penetration, fistula, sinusitis, fistula	-
Degidi et al.	2012	Int J Periodontics Restorative Dent	Double (10)	Full-arch (10)-Partial (0)	12 m	100–100%	-	-
Duarte et al.	2007	Clin Implant Dent Relat Res	Quad (12)	Full-arch (12)-Partial (0)	30 m	97.91%	Osseointegration failure, sinusitis, implant mobility, pain	1 implant
Malevez et al.	2004	Clin Oral Implants Res	Double (55)	Full-arch (55)-Partial (0)	6-48 m	100–91.75%	Sinusitis	-
Maló et al.	2014	Eur J Oral Implantol	Single (73) Double (214), Triple (14) Quad (51)	Full-arch (352)-Partial (0)	6–84 m	98.2–97-9	Sinusitis, implant mobility	14 implants
Maló et al.	2015	Clin Implant Dent Relat Res	Single (8) Double (18) Triple (5) Quad (8)	Full-arch (39)-Partial (0)	36 m	100–100%	Sinusitis, implant mobility, fistula	-
Migliorança et al.	2012	Int J Oral Maxillofac Surg		Full-arch (21)-Partial (0)	96 m	97.50%	Implant mobility	1 implant
Mozzati et al.	2008	Int J Oral Maxillofac Implants	Double (7)	Full-arch (7)-Partial (0)	24 m	100–100%	-	-
Neugarten et al.	2017	Int J Oral Maxillofac Implants	Quad (28)	Full-arch (28)-Partial (0)	54 m	96%-/	Osseointegration failure	4 implants
Pellicer-Chover et al.	2016	Med Oral Patol Oral Cir Bucal	Double (22)	Full-arch (22)-Partial (0)	12 m	97.7–97.8%	-	-
Petrungaro et al.	2020	Compend Contin Educ Dent	Single (134) Double (79) Quad (40)	Full-arch (234)-Partial (15)	60 m	97.6–97.7%	-	11 implants
Sartori et al.	2012	J Oral Maxillofac Surg	Double (16) Quad (3)	Full-arch (16)-Partial (0)	48 m	100–100%		
Stiévenart et al.	2010	Int J Oral Maxillofac Surg	Quad (20)	Full-arch (20)-Partial (0)	6-40 m	96%	Osseointegration failure, sinusitis, implant mobility, pain	3 implants
Urgell et al.	2008	Med Oral Patol Oral Cir Bucal	Single (7) Double (47)	Full-arch (54)-Partial (0)	48 months	96.04–93.22%	Osseointegration failure, sinusitis, implant mobility, pain	4 implants
Zwahlen et al.	2006	Int J Oral Maxillofac Implants	Single (2) Double (15)	Full-arch (18)-Partial (0)	8 m	94.1%-/	Sinusitis, implant mobility, pain, fistula	2 implants

**Table 4:** Summary of the papers evaluated according to the study design, patients treated, zygomatic and regular screws positioned and implant loading protocol.

Authors	Year	Journal	Study	Patients	Zygomatic Implants	Regular Implants	Delayed Loading Zygomatic Implants	Immediate Loading Zygomatic Implants
Agliardi et al.	2017	Int J Oral Maxillofac Surg	P	15	42	18	-	47
Ahlgren et al.	2006	Int J Oral Maxillofac Implants	R	13	25	26	25	-
Aparicio et al.	2010	Clin Implant Dent Relat Res	P	20	36	104	36	-
Aparicio et al.	2006	Clin Implant Dent Relat Res	P	69	131	304	131	-
Aparicio et al.	2010	Clin Implant Dent Relat Res	R	25	47	129	-	47
Aparicio et al.	2014	Clin Implant Dent Relat Res	R	22	41	131	41	-
Balshi et al.	2009	Int J Oral Maxillofac Implants	R	56	110	391	-	110
Becktor et al.	2005	Clin Implant Dent Relat Res	P	16	31	74	31	-
Bedrossian et al.	2006	Int J Oral Maxillofac Implants	P	14	28	55	-	28
Bedrossian et al.	2003	Int J Oral Maxillofac Implants	R	22	44	80	44	-
Butura et al.	2014	Int J Oral Maxillofac Implants	R	15	40	27	-	40
Chana et al.	2018	Int J Oral Maxillofac Implants	R	45	88	180	88	-
Coppede et al.	2017	Clin Implant Dent Relat Res	P	42	94	179	16	78
Davò et al.	2018	Eur J Oral Implantol	P	35	131	237	-	131
Davó et al.	2013	Eur J Oral Implantol	P	42	81	140	-	81
Davó et al.	2010	Eur J Oral Implantol	P	17	68	-	-	68
Davó et al.	2008	Eur J Oral Implantol	P	42	81	140	-	81
Davó et al.	2013	Eur J Oral Implantol	P	17	68	-	-	68
Degidi et al.	2012	Int J Periodontics Restorative Dent	P	10	20	20	-	20
Duarte et al.	2007	Clin Implant Dent Relat Res	P	12	48	-	-	48
Malevez et al.	2004	Clin Oral Implants Res	R	55	103	194	103	-
Maló et al.	2014	Eur J Oral Implantol	R	352	747	795	-	747
Maló et al.	2015	Clin Implant Dent Relat Res	R	39	92	77	-	92
Migliorança et al.	2012	Int J Oral Maxillofac Surg	P	21	40	74	-	40
Mozzati et al.	2008	Int J Oral Maxillofac Implants	P	7	14	34	-	14
Neugarten et al.	2017	Int J Oral Maxillofac Implants	R	28	105	-	-	105
Pellicer-Chover et al.	2016	Med Oral Patol Oral Cir Bucal	R	22	44	94	44	-
Petrungaro et al.	2020	Compend Contin Educ Dent	P	249	452	360	249	-
Sartori et al.	2012	J Oral Maxillofac Surg	R	16	37	58	16	-
Stiévenart et al.	2010	Int J Oral Maxillofac Surg	P	20	80	-	40	40
Urgell et al.	2008	Med Oral Patol Oral Cir Bucal	R	54	101	221	101	-
Zwahlen et al.	2006	Int J Oral Maxillofac Implants	R	18	34	42	-	34

## Data Availability

All experimental data to support the findings of this study are available contacting the corresponding author upon request. The authors have annotated the entire data building process and empirical techniques presented in the paper.

## References

[1] Wagner F., Dvorak G., Nemec S., Pietschmann P., Figl M., Seemann R. (2016). A principal components analysis: How pneumatization and edentulism contribute to maxillary atrophy. Oral Dis..

[2] Cawood J., Howell R. (1988). A classification of the edentulous jaws. Int. J. Oral Maxillofac. Surg..

[3] Unger J.W., Ellinger C.W., Gunsolley J.C. (1992). An analysis of the effect of mandibular length on residual ridge loss in the edentulous patient. J. Prosthet. Dent..

[4] Al-Thobity A., Wolfinger G., Balshi S., Flinton R., Balshi T. (2014). Zygomatic implants as a rehabilitation approach for a severely deficient maxilla. Int. J. Oral Maxillofac. Implant..

[5] Hernández-Alfaro F., Ragucci G.M.M., Méndez-Manjón I., Giralt-Hernando M., Guijarro-Martínez R., Sicilia-Blanco P., Ventura-Martínez N., Valls-Ontañón A. (2019). Rehabilitation of the severely atrophic maxilla using LeFort I maxillary advancement and simultaneous zygoma implant placement: Proof of concept. Int. J. Oral Implantol..

[6] Comuzzi L., Tumedei M., Piattelli A., Iezzi G. (2019). Short vs. standard length cone morse connection implants: An in vitro pilot study in low density polyurethane foam. Symmetry.

[7] Comuzzi L., Tumedei M., Pontes A.E., Piattelli A., Iezzi G. (2020). Primary stability of dental implants in low-density (10 and 20 pcf) polyurethane foam blocks: Conical vs cylindrical implants. Int. J. Environ. Res. Public Health.

[8] Tumedei M., Piattelli A., Degidi M., Mangano C., Iezzi G. (2020). A Narrative review of the histological and histomorphometrical evaluation of the peri-implant bone in loaded and unloaded dental implants. a 30-year experience (1988–2018). Int. J. Environ. Res. Public Health.

[9] Fanali S., Tumedei M., Pignatelli P., Inchingolo F., Pennacchietti P., Pace G., Piattelli A. (2020). Implant primary stability with an osteocondensation drilling protocol in different density polyurethane blocks. Comput. Methods Biomech. Biomed. Eng..

[10] Fujiwara S., Kato S., Bengazi F., Velez J.U., Tumedei M., Kotsu M., Botticelli D. (2021). Healing at implants installed in osteotomies prepared either with a piezoelectric device or drills: An experimental study in dogs. Oral Maxillofac. Surg..

[11] Kotsu M., Urbizo Velez J., Bengazi F., Tumedei M., Fujiwara S., Kato S., Botticelli D. (2021). Healing at implants installed from ~70- to <10-Ncm insertion torques: An experimental study in dogs. Oral Maxillofac. Surg..

[12] De Moraes P., Olate S., Nóbilo M.D.A., Asprino L., De Moraes M., Barbosa J.D.A. (2016). Maxillary “All-On-Four” treatment using zygomatic implants. A mechanical analysis. Rev. Stomatol. Chir. Maxillofac. Chir. Orale.

[13] Ferreira E.J., Kuabara M.R., Gulinelli J.L. (2010). “All-on-four” concept and immediate loading for simultaneous rehabilitation of the atrophic maxilla and mandible with conventional and zygomatic implants. Br. J. Oral Maxillofac. Surg..

[14] Jensen O.T., Adams M.W., Butura C., Galindo D.F. (2015). Maxillary V-4: Four implant treatment for maxillary atrophy with dental implants fixed apically at the vomer-nasal crest, lateral pyriform rim, and zygoma for immediate function. Report on 44 patients followed from 1 to 3 years. J. Prosthet. Dent..

[15] Malo P., de Araújo Nobre M., Lopes A., Moss S.M., Molina G.J. (2011). A longitudinal study of the survival of All-on-4 implants in the mandible with up to 10 years of follow-up. J. Am. Dent. Assoc..

[16] Bavetta G., Bavetta G., Randazzo V., Cavataio A., Paderni C., Grassia V., Dipalma G., Isacco C.G., Scarano A., De Vito D. (2019). A retrospective study on insertion torque and Implant Stability Quotient (ISQ) as stability parameters for immediate loading of implants in fresh extraction sockets. BioMed. Res. Int..

[17] Balan I., Di Girolamo M., Lauritano D., Carinci F. (2017). Treatment of severe atrophic maxilla with zygomatic implants: A case series. Oral Implantol..

[18] Scarano A., Conte R., Murmura G., Lorusso F., Harrath A.H. (2019). Satisfaction grade assessment of patients treated with zygomatic implants with self-tapping apex and machined body. J. Biol. Regul. Homeost. Agents.

[19] Scarano A., Lorusso F., Arcangelo M., D’Arcangelo C., Celletti R., De Oliveira P.S. (2018). Lateral sinus floor elevation performed with trapezoidal and modified triangular flap designs: A randomized pilot study of post-operative pain using thermal infrared imaging. Int. J. Environ. Res. Public Health.

[20] Scarano A., Murmura G., Mastrangelo F., Lorusso F., Greco Lucchina A., Carinci F. (2018). A novel technique to prevent sinus membrane collapse during maxillary sinus floor augmentation without bone graft: Technical note. J. Biol. Regul. Homeost. Agents.

[21] Ito Z., Matsuyama Y., Sakai Y., Imagama S., Wakao N., Ando K., Hirano K., Tauchi R., Muramoto A., Matsui H. (2010). Bone union rate with autologous iliac bone versus local bone graft in posterior lumbar interbody fusion. Spine.

[22] De Carvalho F.A., Ponzoni D., Vedovatto E., de Carvalho P.S.P. (2020). Remodeling of calvarial graft in increased atrophic maxillary thickness. A prospective clinical study. Clin. Implant Dent. Relat. Res..

[23] Aghaloo T.L., Tencati E., Hadaya D. (2019). Biomimetic enhancement of bone graft reconstruction. Oral Maxillofac. Surg. Clin. N. Am..

[24] Bertolai R., Aversa A., Catelani C., Rossi A., Giannini D. (2015). Treatment of extreme maxillary atrophy with Zygoma implants. Minerva Stomatol..

[25] Karlan M.S., Cassisi N.J. (1979). Fractures of the zygoma: A geometric, biomechanical, and surgical analysis. Arch. Otolaryngol. Head Neck Surg..

[26] Uchida Y., Goto M., Katsuki T., Akiyoshi T. (2001). Measurement of the maxilla and zygoma as an aid in installing zygomatic implants. J. Oral Maxillofac. Surg..

[27] Nkenke E., Hahn M., Lell M., Wiltfang J., Schultze-Mosgau S., Stech B., Radespiel-Tröger M., Neukam F.W. (2003). Anatomic site evaluation of the zygomatic bone for dental implant placement: Zygomatic bone for dental implants. Clin. Oral Implant. Res..

[28] Romeed S.A., Malik R., Dunne S.M. (2014). Zygomatic implants: The impact of zygoma bone support on biomechanics. J. Oral Implant..

[29] Davó R., David L. (2019). Quad zygoma: Technique and realities. Oral Maxillofac. Surg. Clin. N. Am..

[30] Chrcanovic B.R., Abreu M.H.N.G. (2012). Survival and complications of zygomatic implants: A systematic review. Oral Maxillofac. Surg..

[31] Chrcanovic B.R., Albrektsson T., Wennerberg A. (2016). Survival and complications of zygomatic implants: An updated systematic review. J. Oral Maxillofac. Surg..

[32] Moher D., Liberati A., Tetzlaff J., Altman D.G. (2010). Preferred reporting items for systematic reviews and meta-analyses: The PRISMA statement. Int. J. Surg..

[33] Agliardi E., Romeo D., Panigatti S., de Araújo Nobre M., Maló P. (2017). Immediate full-arch rehabilitation of the severely atrophic maxilla supported by zygomatic implants: A prospective clinical study with minimum follow-up of 6 years. Int. J. Oral Maxillofac. Surg..

[34] Ahlgren F., Størksen K., Tornes K. (2006). A study of 25 zygomatic dental implants with 11 to 49 months’ follow-up after loading. Int. J. Oral Maxillofac. Implant..

[35] Aparicio C., Ouazzani W., Aparicio A., Fortes V., Muela R., Pascual A., Codesal M., Barluenga N., Franch M. (2010). Immediate/early loading of zygomatic implants: Clinical experiences after 2 to 5 years of follow-up. Clin. Implant Dent. Relat. Res..

[36] Aparicio C., Ouazzani W., Aparicio A., Fortes V., Muela R., Pascual A., Codesal M., Barluenga N., Manresa C., Franch M. (2010). Extrasinus zygomatic implants: Three year experience from a new surgical approach for patients with pronounced buccal concavities in the edentulous maxilla. Clin. Implant Dent. Relat. Res..

[37] Aparicio C., Ouazzani W., Garcia R., Arevalo X., Muela R., Fortes V. (2006). A prospective clinical study on titanium implants in the zygomatic arch for prosthetic rehabilitation of the atrophic edentulous maxilla with a follow-up of 6 months to 5 years. Clin. Implant Dent. Relat. Res..

[38] Aparicio C., Manresa C., Francisco K., Ouazzani W., Claros P., Potau J.M., Aparicio A. (2014). The long-term use of zygomatic implants: A 10-year clinical and radiographic report. Clin. Implant Dent. Relat. Res..

[39] Balshi S.F., Wolfinger G.J., Balshi T.J. (2009). A retrospective analysis of 110 zygomatic implants in a single-stage immediate loading protocol. Int. J. Oral Maxillofac. Implant..

[40] Becktor J.P., Isaksson S., Abrahamsson P., Sennerby L. (2005). Evaluation of 31 zygomatic implants and 74 regular dental implants used in 16 patients for prosthetic reconstruction of the atrophic maxilla with cross-arch fixed bridges. Clin. Implant Dent. Relat. Res..

[41] Bedrossian E., Stumpel L., Beckely M.L., Indresano T., Indersano T. (2002). The zygomatic implant: Preliminary data on treatment of severely resorbed maxillae. A clinical report. Int. J. Oral Maxillofac. Implant..

[42] Bedrossian E., Rangert B., Stumpel L., Indresano T. (2006). Immediate function with the zygomatic implant: A graftless solution for the patient with mild to advanced atrophy of the maxilla. Int. J. Oral Maxillofac. Implant..

[43] Butura C.C., Galindo D.F. (2014). Combined immediate loading of zygomatic and mandibular implants: A preliminary 2-year report of 19 patients. Int. J. Oral Maxillofac. Implant..

[44] Chana H., Smith G., Bansal H., Zahra D. (2019). A retrospective cohort study of the survival rate of 88 zygomatic implants placed over an 18-year period. Int. J. Oral Maxillofac. Implant..

[45] Coppedê A., De Mayo T., de Sá Zamperlini M., Amorin R., De Pádua A.P.A.T., Shibli J.A. (2017). Three-year clinical prospective follow-up of extrasinus zygomatic implants for the rehabilitation of the atrophic maxilla. Clin. Implant Dent. Relat. Res..

[46] Davó R., Felice P., Pistilli R., Barausse C., Marti-Pages C., Ferrer-Fuertes A., Ippolito D.R., Esposito M. (2018). Immediately loaded zygomatic implants vs conventional dental implants in augmented atrophic maxillae: 1-year post-loading results from a multicentre randomised controlled trial. Eur. J. Oral Implantol..

[47] Davó R., Malevez C., Pons O. (2013). Immediately loaded zygomatic implants: A 5-year prospective study. Eur. J. Oral Implantol..

[48] Davó R., Pons O. (2013). Prostheses supported by four immediately loaded zygomatic implants: A 3-year prospective study. Eur. J. Oral Implantol..

[49] Davo R., Pons O., Rojas J., Carpio E. (2010). Immediate function of four zygomatic implants: A 1-year report of a prospective study. Eur. J. Oral Implantol..

[50] Davó R., Malevez C., Rojas J., Rodríguez J., Regolf J. (2008). Clinical outcome of 42 patients treated with 81 immediately loaded zygomatic implants: A 12- to 42-month retrospective study. Eur. J. Oral Implantol..

[51] Degidi M., Nardi D., Piattelli A., Malevez C. (2012). Immediate loading of zygomatic implants using the intraoral welding technique: A 12-month case series. Int. J. Periodontics Restor. Dent..

[52] Duarte L.R., Filho H.N., Francischone C.E., Peredo L.G., Brånemark P.-I. (2007). The establishment of a protocol for the total rehabilitation of atrophic maxillae employing four zygomatic fixtures in an immediate loading system—A 30-month clinical and radiographic follow-Up. Clin. Implant Dent. Relat. Res..

[53] Malevez C., Abarca M.M., Durdu F.F., Daelemans P. (2004). Clinical outcome of 103 consecutive zygomatic implants: A 6–48 months follow-up study. Clin. Oral Implant. Res..

[54] Maló P., Nobre M.D.A., Lopes A., Ferro A., Moss S. (2014). Five-year outcome of a retrospective cohort study on the rehabilitation of completely edentulous atrophic maxillae with immediately loaded zygomatic implants placed extra-maxillary. Eur. J. Oral Implantol..

[55] Maló P., de Araújo Nobre M., Lopes A., Ferro A., Moss S. (2015). Extramaxillary surgical technique: Clinical outcome of 352 patients rehabilitated with 747 zygomatic implants with a follow-up between 6 months and 7 years. Clin. Implant Dent. Relat. Res..

[56] Migliorança R.M., Sotto-Maior B.S., Senna P.M., Francischone C.E., Del Bel Cury A.A. (2012). Immediate occlusal loading of extrasinus zygomatic implants: A prospective cohort study with a follow-up period of 8 years. Int. J. Oral Maxillofac. Surg..

[57] Mozzati M., Monfrin S.B., Pedretti G., Schierano G., Bassi F. (2008). Immediate loading of maxillary fixed prostheses retained by zygomatic and conventional implants: 24-month preliminary data for a series of clinical case reports. Int. J. Oral Maxillofac. Implant..

[58] Neugarten J., Tuminelli F., Walter L. (2017). Two bilateral zygomatic implants placed and immediately loaded: A retrospective chart review with up-to-54-month follow-up. Int. J. Oral Maxillofac. Implant..

[59] Pellicer-Chover H., Cervera-Ballester J., Penarrocha-Oltra D., Bagan L., Penarrocha-Diago M. (2016). Influence of the prosthetic arm length (palatal position) of zygomatic implants upon patient satisfaction. Med. Oral Patol. Oral Cir. Bucal.

[60] Petrungaro P.S., Gonzales S., Villegas C., Yousef J., Arango A. (2020). A Retrospective study of a multi-center case series of 452 zygomatic implants placed over 5 years for treatment of severe maxillary atrophy. Compend. Contin. Educ. Dent..

[61] Sartori E.M., Padovan L.E.M., de Mattias Sartori I.A., Ribeiro P.D., Carvalho A.C.G.D.S., Goiato M.C. (2012). Evaluation of satisfaction of patients rehabilitated with zygomatic fixtures. J. Oral Maxillofac. Surg..

[62] Stiévenart M., Malevez C. (2010). Rehabilitation of totally atrophied maxilla by means of four zygomatic implants and fixed prosthesis: A 6–40-month follow-up. Int. J. Oral Maxillofac. Surg..

[63] Urgell J.P., Gutiérrez V.R., Escoda C.G.G. (2008). Rehabilitation of atrophic maxilla: A review of 101 zygomatic implants. Med. Oral Patol. Oral Cir. Bucal.

[64] Zwahlen R.A., Grätz K.W., Oechslin C.K., Studer S.P. (2006). Survival rate of zygomatic implants in atrophic or partially resected maxillae prior to functional loading: A retrospective clinical report. Int. J. Oral Maxillofac. Implant..

[65] Gehrke S.A., Mazón P., Del Fabbro M., Tumedei M., Aramburú Júnior J., Pérez-Díaz L., De Aza P.N. (2019). Histological and Histomorphometric Analyses of Two Bovine Bone Blocks Implanted in Rabbit Calvaria. Symmetry.

[66] Javed F., Romanos G.E. (2010). The role of primary stability for successful immediate loading of dental implants. A literature review. J. Dent..

[67] Scarano A., De Oliveira P.S., Traini T., Lorusso F. (2018). Sinus Membrane Elevation with Heterologous Cortical Lamina: A Randomized Study of a New Surgical Technique for Maxillary Sinus Floor Augmentation without Bone Graft. Materials.

[68] Scarano A., Inchingolo F., Murmura G., Traini T., Piattelli A., Lorusso F. (2018). Three-Dimensional Architecture and Mechanical Properties of Bovine Bone Mixed with Autologous Platelet Liquid, Blood, or Physiological Water: An In Vitro Study. Int. J. Mol. Sci..

[69] Veltri M., González-Martín O., Belser U.C. (2014). Influence of simulated bone-implant contact and implant diameter on secondary stability: A resonance frequency in vitro study. Clin. Oral Implant. Res..

[70] Hsu J.-T., Shen Y.-W., Kuo C.-W., Wang R.-T., Fuh L.-J., Huang H.-L. (2017). Impacts of 3D bone-to- implant contact and implant diameter on primary stability of dental implant. J. Formos. Med. Assoc..

[71] Chappuis V., Rahman L., Buser R., Janner S., Belser U., Buser D. (2017). Effectiveness of contour augmentation with guided bone regeneration: 10-year results. J. Dent. Res..

[72] González-Martín O., Lee E., Weisgold A., Veltri M., Su H. (2020). Contour management of implant restorations for optimal emergence profiles: Guidelines for immediate and delayed provisional restorations. Int. J. Periodontics Restor. Dent..

[73] Jensen O.T., Adams M.W., Smith E. (2014). Paranasal bone: The prime factor affecting the decision to use transsinus vs zygomatic implants for biomechanical support for immediate function in maxillary dental implant reconstruction. Int. J. Oral Maxillofac. Implant..

[74] Gümrükçü Z. (2019). Biomechanical evaluation of zygomatic implant use in patients with different buccal maxillary defect levels. Int. J. Oral Maxillofac. Implant..

[75] Al-Nawas B., Wegener J., Bender C., Wagner W. (2004). Critical soft tissue parameters of the zygomatic implant. J. Clin. Periodontol..

[76] Peñarrocha-Diago M., Bernabeu-Mira J.C., Fernández-Ruíz A., Aparicio C., Peñarrocha-Oltra D. (2020). Bone regeneration and soft tissue enhancement around zygomatic implants: Retrospective case series. Materials.

[77] Brånemark P., Gröndahl K., Öhrnell L., Nilsson P., Petruson B., Svensson B., Engstrand P., Nannmark U. (2004). Zygoma fixture in the management of advanced atrophy of the maxilla: Technique and long-term results. Scand. J. Plast. Reconstr. Surg. Hand Surg..

[78] Albrektsson T., Wennerberg A. (2019). On osseointegration in relation to implant surfaces. Clin. Implant Dent. Relat. Res..

[79] Scarano A., Piattelli A., Quaranta A., Lorusso F. (2017). Bone response to two dental implants with different sandblasted/acid-etched implant surfaces: A histological and histomorphometrical study in rabbits. BioMed Res. Int..

